# Treatment of Cancer-Associated Thrombosis: Beyond HOKUSAI

**DOI:** 10.1055/s-0039-1696659

**Published:** 2019-09-16

**Authors:** Isabelle Mahé, Ismaïl Elalamy, Grigoris T. Gerotziafas, Philippe Girard

**Affiliations:** 1 Université de Paris, Innovations Thérapeutiques en Hémostase, INSERM, Paris, France; 2 Service de médecine Interne, AH-HP, Hôpital Louis Mourier, Colombes, Université de Paris, France; 3 F-CRIN INNOVTE, Saint Etienne, France; 4 Hematology and Thrombosis Center, Tenon University Hospital, Sorbonne University, INSERM U938, Paris, France; 5 Department of Obstetrics and Gynecology, The First I.M. Sechenov Moscow State Medical University, Moscow, Russia; 6 Research Group “Cancer, Haemostasis and Angiogenesis,” INSERM UMR_S 938, Centre de Recherche Saint-Antoine, Faculty of Medicine, Institut Universitaire de Cancérologie, Sorbonne Universities, Paris, France; 7 Service d’Hématologie Biologique Hôpital Tenon, Hôpitaux Universitaires de l’Est Parisien, APHP.6, Paris, France; 8 Institut du Thorax Curie-Montsouris, l’Institut Mutualiste Montsouris, Paris, France

**Keywords:** cancer-associated thrombosis, direct oral anticoagulants, low-molecular-weight heparins

## Abstract

Direct oral anticoagulants (DOACs) represent an attractive alternative to low-molecular-weight heparins (LMWHs) for the long-term treatment of cancer-associated thrombosis (CT) since they avoid the burden of daily injections. Analyses in subgroups of cancer patients from large randomized trials suggested that DOACs were at least as effective as vitamin K antagonists, while indirect comparisons suggested that DOACs' efficacy and safety profile were comparable to those of LMWHs. In the randomized controlled HOKUSAI-VTE Cancer study, currently the only completed phase III trial on DOACs in CT patients, edoxaban was shown noninferior to dalteparin on the composite primary endpoint of time to first recurrent venous thromboembolism or major bleeding during the 12 months after randomization. Study results suggest that both agents had comparable benefit/risk ratio in patients with CT. Even though this conclusion was valid from a strict statistical viewpoint, it was potentially misleading when interpreting benefit/risk ratios. Besides the obvious heterogeneity of the study population (e.g., 23% of patients no longer had cancer) and significantly different treatment durations between arms, secondary outcomes for efficacy were in favor of edoxaban for recurrent deep-vein thrombosis but not for recurrent pulmonary embolism, and major bleeding episodes were significantly more frequent in the edoxaban group, with an excess of gastrointestinal (GI) bleeding episodes observed mainly but not only in patients with GI cancers. More research is needed regarding specific patients' profiles, cancer types, and treatment period to better clarify the respective roles of DOACs and LMWHs in CT patients.

## Introduction

International treatment guidelines recommend low-molecular-weight heparins (LMWHs) as the first choice for the long-term treatment of cancer-associated thrombosis (CT). 1 2 3 However this therapeutic strategy faces substantial limitations mainly due to the need of daily subcutaneous injections which are not well accepted by all patients for the recommended 6-month or even 12-month treatment duration, 4 5 even though clinicians tend to overestimate this issue. 6 Also, patients prefer an anticoagulant that does not interfere with their cancer treatment, suggesting the primacy of cancer disease over venous thromboembolism (VTE). 7

The introduction of direct oral anticoagulants (DOACs) has raised strong expectations since they potentially represent an attractive alternative to vitamin K antagonists (VKAs) and LMWHs as they do not require dose adjustment after laboratory monitoring and they avoid the burden of daily injections especially in the context of the recommended treatment duration. 8 9 10 11 12

Based on the results of large noninferiority randomized controlled trials (RCTs), DOACs were approved for the treatment of VTE. 13 Post hoc analyses of these studies suggested that DOACs were at least as effective and safe as VKA in the subgroups of patients with CT, while indirect comparisons suggested that LMWHs and DOACs were either of comparable efficacy and safety 14 or found that bleeding rates were higher with DOACs compared with LMWHs. 15 Nonetheless, several noncontrolled prospective and retrospective cohort studies provided further support for DOACs as attractive alternatives to LMWHs in terms of efficacy and safety clinical outcomes in CT patients. 16 17 18 19 20

The HOKUSAI-VTE Cancer study 21 is the first significant contribution for the evaluation of DOACs for the long-term treatment of CT; it is currently the only phase III prospective RCT comparing DOACs and LMWHs for the long-term treatment of CT. The study was well conducted, properly powered, and demonstrated the noninferiority of edoxaban to dalteparin. However, the unusual combination of efficacy and safety in the primary composite endpoint made the outcome difficult to interpret in terms of clinical relevance regarding the balance between safety and efficacy. The aim of our manuscript is to assess whether the HOKUSAI-VTE Cancer study design and results provide meaningful evidence for a new alternative to LMWH for the long-term treatment of patients with CT and to identify methodology issues that should improve the design of future studies.

## HOKUSAI-VTE Cancer Main Outcomes

This phase III noninferiority RCT compared the orally active specific and direct inhibitor of factor Xa, edoxaban, to LMWH dalteparin for the long-term treatment of patients with CT. It is the first well-conducted published trial on DOACs with an adequate sample size ( *N* = 1,050) and sufficient follow-up of 12 months. The patients' clinical characteristics at inclusion were well balanced between groups. The only significant difference between treatment arms that must be noticed before analyzing the results is treatment duration, which was significantly shorter in the dalteparin group (median: 184 days) than in the edoxaban group (median: 211 days, *p* = 0.01).

Edoxaban was shown noninferior to dalteparin on the composite primary endpoint of “recurrent VTE or major bleeding during the 12 months after randomization, regardless of treatment duration.” The primary outcome occurred in 67 of the 522 patients (12.8%) in the edoxaban group and in 71 of the 524 patients (13.5%) in the dalteparin group (hazard ratio: 0.97; 95% confidence interval [CI]: 0.70–1.36; *p* = 0.006 for noninferiority; *p* = 0.87 for superiority).

Taken separately, recurrent VTE occurred in 41 patients (7.9%) in the edoxaban group and in 59 patients (11.3%) in the dalteparin group (difference in risk: −3.4 percentage points [95% CI: −7.0 to 0.2]; hazard ratio: 0.71 [95% CI: 0.48–1.06; *p* = 0.09]) while major bleeding occurred in 36 patients (6.9%) in the edoxaban group and in 21 patients (4.0%) in the dalteparin group (difference in risk: 2.9 percentage points [95% CI: 0.1–5.6]; hazard ratio: 1.77 [95% CI: 1.03–3.04; *p* = 0.04]), revealing a statistically significant increase in the rate of major bleeding episodes in edoxaban-treated patients compared with those treated with dalteparin.

## HOKUSAI-VTE Cancer Protocol Relevance

### Patient Population

The patient population appears heterogeneous. Almost all patients had “active cancer” (>97%), half of them had metastatic disease (>52%), and 71% were receiving antineoplastic therapy. However, the definition of “active cancer,” which was also used in other recent trials of CT patients, 22 23 allowed the selection of patients whose cancer was “diagnosed within the previous 6 months,” and the HOKUSAI investigators even added the criterion of cancer “diagnosed within the previous 2 years.” This difference of 6 or 24 months in the interval between cancer diagnosis and enrolment in the study is the likeliest explanation for the fact that 239 (23%) of the 1,050 patients had their cancer “cured” at the time of randomization, an interesting characteristic for the so-called CT patients. 21 Strict criteria for the definition of active cancer as proposed by Kearon et al 24 allowing the selection of a more homogeneous study population were not published at the time of study initiation. Therefore, the study may appear underpowered to conclude on the efficacy and safety of edoxaban compared with dalteparin in CT patients with “true” active cancer, i.e., the real population of interest.

### Relevance of Study Outcomes

VTE recurrence has always been considered as the unique primary efficacy endpoint while major bleeding was a secondary safety endpoint in the main RCTs comparing anticoagulants for the treatment of patients with CT. 22 25 26 27 28 29 30 This allowed an adequate separate assessment of the components of the benefit/risk ratio which is the basis to establish clinical practice guidelines.

The selection of the primary composite endpoint combining recurrent VTE and major bleeding in the HOKUSAI-VTE Cancer helped to ensure adequate statistical power. However, the conclusion of noninferiority based on an endpoint mixing efficacy and safety may appear of limited clinical relevance as it suggests that both agents have similar benefit–risk profile. Instead, edoxaban use is associated with a (nonsignificant) a lower VTE recurrence rate, but a (significant) higher risk of major bleeding as compared with dalteparin. In other words, the composite endpoint does not allow appreciating the safety price, i.e., the excess in bleeding risk needed to pay to avoid VTE recurrences.

The composite primary endpoint was set to be measured at 12 months. Yet patients were supposed to receive the full anticoagulant treatment (dalteparin or edoxaban) for 6 months, while the treatment beyond 6 months was left to the investigator's judgment as it is often the case in usual clinical practice. However, this approach together with a loss of power between 6 and 12 months given the remaining small sample size questions the 12-month endpoint validity given the substantial imbalance between treatment arms as only 200 (38.3%) and 154 (29.4%) patients completed 12 months of treatment with edoxaban and dalteparin, respectively.

### VTE Recurrence

Although a trend toward a lower VTE recurrence rate with edoxaban was observed ( *p* = 0.09), this trend was driven only by recurrent DVTs, as there was no difference in recurrent pulmonary embolism rates between the two compounds (5.2 vs. 5.3%). This point deserves consideration when case fatality is a concern. More importantly, the careful analysis of the Kaplan–Meier curves shows that the recurrence rate is absolutely identical within the first 3 months of treatment, i.e., when the same proportion of patients in both arms actually receive the study drugs (73.4 and 73.9% in the edoxaban and dalteparin arms, respectively). 21 Between 3 and 6 months, when the curves separate, more patients discontinue their treatment in the dalteparin arm than in the edoxaban arm (102 and 80 patients, respectively), a trend that persists up to the end of the study period and becomes statistically significant. There is no information about the anticoagulant received after discontinuation of the study drug and there are differences between arms in treatment duration while the respective rates of VTE recurrences on and off treatment (per protocol analysis) are unfortunately unknown thus making it impossible to draw definite conclusions on differences in efficacy between arms.

### Major Bleeding

The selection of dalteparin as a comparator was appropriate as the drug is a standard of care approved for the long-term treatment of patients with CT. An excess of bleeding with edoxaban was shown early in the study in the Kaplan–Meier estimate of cumulative major bleeding rates. The median number of days from randomization to a major bleeding event was 61 (interquartile range [IQR]: 23–174 days) in the edoxaban group, and 91 (IQR: 37–133 days) in the dalteparin group. Furthermore, unlike VTE recurrences, the Kaplan–Meier curves separate immediately after inclusion, supporting a difference in bleeding risk between edoxaban and dalteparin. In addition, in patients with two or more bleeding risk factors, major bleeding occurred in 7.4% receiving edoxaban, and in 3.6% of patients treated with dalteparin, indicating that in vulnerable patients dalteparin tended to be safer than edoxaban. 31

The difference in bleeding rates was due to a higher incidence of gastrointestinal (GI) bleeding with edoxaban (3.8%) compared with dalteparin (1.1%), while the proportion of patients with GI cancer at baseline was comparable in both treatment arms (116 [22.2%] patients and 100 [19.1%] patients in the edoxaban and dalteparin arms, respectively). Subgroup analyses confirmed that patients with GI cancer were more likely to have an increased risk of bleeding during treatment with edoxaban than with dalteparin ( *p* = 0.02 for interaction in the safety population) while the increase in upper GI major bleeding occurred mainly in patients who had entered the trial with GI cancer. However, it is important to underline that the upper GI bleeds in the edoxaban group were evenly distributed among the various types of GI cancer. Lower GI bleeds mostly occurred in patients with colorectal cancer in both treatment groups. 31 This indicates that in edoxaban-treated patients the risk of major bleeding at the GI track is not dictated by tumor localization. This is in the same line with the results from a large RCT of 22,000 patients with atrial fibrillation and without active cancer in which the annualized rate of major GI bleeding was significantly higher with edoxaban 60 mg daily than with warfarin (1.51 vs. 1.23%, 1.23 [1.02–1.50], *p* = 0.03), whereas the overall rate of major bleeding was significantly lower with edoxaban compared with warfarin (2.75 vs. 3.43%, 0.80 [0.71–0.91], *p* < 0.001). 32 GI bleeding with edoxaban therefore not only appears to be related to the initial cancer diagnosis but also suggests a direct effect of edoxaban on the GI tract. The mechanism of this effect needs further clarification.

In the Select-D Pilot trial, 23 406 CT patients were treated for 6 months with either rivaroxaban or dalteparin. Treatment with rivaroxaban was associated with a nominally lower VTE recurrence (4%; 95% CI: 2–9%) compared with dalteparin (11%; 95% CI: 7–17%), and major bleeding rates were similar across treatment arms. Taken together, major bleeds and clinically relevant nonmajor bleeds (CRNMBs) were markedly more frequent in the rivaroxaban arm (17%; 95% CI: 12–22%) than in the dalteparin arm (5%; 95% CI: 12–22%). However, Select-D trial results should be interpreted with caution given the pilot design limitations. A meta-analysis of the Select-D and HOKUSAI-VTE Cancer trials confirmed that anti-Xa DOACs tended to be more effective than LMWH in reducing VTE recurrence at the expense of a significant increase in major bleeding events and a trend toward more CRNMB with DOACs, especially in patients with GI cancer who may be at the highest risk for bleeding. 33 Interestingly, the recently reported phase IV ADAM trial failed to demonstrate a decreased risk of bleeding with apixaban as compared with dalteparin in CT patients. 34 The results of the ongoing phase III CARAVAGGIO study 35 will help clarify the benefit–risk of the DOAC apixaban in patients with CT.

### Impact on Clinical Practice

LMWHs remain the standard of care for the treatment of patients with CT and current guidelines, published before the HOKUSAI-VTE Cancer trial results were available, do not have a preference for DOACs 1 even though in many aspects DOACs represent an attractive alternative to LMWH for the treatment of patients with CT as they do not require daily injections or continuous intervention of health professionals which is a source of additional costs.

In view of the clinical data reported to date with edoxaban in CT patients, changes in guidelines may not be easily considered. In trying to lower the bleeding risk, reducing DOAC doses proposed in noncancer patients in the extension program is a challenge in cancer patients as they are at a high risk of both thrombosis and bleeding and there are no data suggesting that dose reduction could benefit safety without compromising efficacy. Based on the updated evidence and the limited experience with LMWH beyond 6 months, treatment guidelines may consider DOACs as a potential alternative in specific cases, with a more precise description of their use in patients with CT. To date, the American College of Chest Physicians suggests LMWH over VKA (Grade 2B), dabigatran (Grade 2C), rivaroxaban (Grade 2C), apixaban (Grade 2C), or edoxaban (Grade 2C) for VTE and cancer. 24 The extrapolation from limited numbers of CT patients in cohorts of CT patients with heterogeneous cancer types is still questionable and requires a more homogeneous evaluation with a more tailored strategy. This is reflected in the International Society on Thrombosis and Haemostasis (ISTH) guidelines, 36 37 which suggest that the risk of bleeding should be individually identified prior to the decision to use either a LMWH or a DOAC. Finally, the recently published American Society of Clinical Oncology guidelines recommend “LMWH, edoxaban, or rivaroxaban for at least 6 months” in CT patients (strength of recommendation: strong), while mentioning the “increase in major bleeding risk with DOACs, particularly observed in GI and potentially genitourinary malignancies.” 38

Finally, the issue of the practical use of DOACs in the context of malignancy in which patients may be exposed to variations of the anticoagulant effect due to drug–drug interactions with antineoplastic treatments and/or comorbidities such as renal insufficiency also needs further evaluation. 39

### Implications in the Conduct of Future Clinical Trials

RCTs on anticoagulants for the treatment of CT conducted since early 2000s are heterogeneous with regard to study design ( Table 1 ). Beside the pilot studies, limitations include a modest sample size, 25 27 40 insufficient treatment duration and/or maintenance during follow-up, 21 26 a composite primary endpoint of VTE recurrence, and major bleeding. 21 25

**Table 1:** Protocol design of randomized control trials on anticoagulants for the treatment of cancer-associated thrombosis

Study (year)	Study drug/comparator	Patients	Methods/statistics	Primary endpoint	Secondary endpoints	Duration (mo)
ONCENOX 39 *N* = 122 (2006)	Enoxaparin/warfarin	Active cancer, acute symptomatic VTE	Pilot feasibility	VTE recurrence	Major and minor bleeding	6
CANTHANOX 25 *N* = 146 (2002)	Enoxaparin/warfarin	Active or treated cancer, PE, and/or DVT	S	Composite of major bleeding or recurrent VTE	Recurrent VTE Major bleeding	3
CLOT 26 *N* = 672 (2003)	Dalteparin/warfarin	Active cancers, acute proximal symptomatic DVT or PE	Phase III/S	Symptomatic recurrence of DVT, PE, or both	Major bleeding Any bleeding	6
LITE CANCER 27 *N* = 200 (2006)	Tinzaparin/warfarin	Cancer, acute proximal DVT	Phase III/S	Recurrent VTE or death	Major and minor bleeding	3
CATCH 22 *N* = 900 (2015)	Tinzaparin/warfarin	Active cancers, acute proximal symptomatic DVT or PE	Phase III/S	VTE recurrence: proximal DVT, PE either symptomatic or incidental	Major bleeding CRNMB	6
SELECT-D 23 *N* = 203 (2018)	Rivaroxaban/dalteparin	All active cancers, proximal DVT, PE, incidental PE	Pilot	VTE recurrence: proximal DVT, PE either symptomatic or incidental, and other sites	Major bleeding CRNMB	6
HOKUSAI-VTE Cancer 21 *N* = 1,046 (2018)	Edoxaban/dalteparin	Active or history of cancer, acute symptomatic or incidental VTE	Phase III/NI	Composite of VTE recurrence (symptomatic or incidental) or major bleeding	VTE recurrence Major bleeding CRNMB	12
CARAVAGGIO 35 *N* = 1,168 (2019)	Apixaban/dalteparin	Active or history of cancer, symptomatic or incidental proximal DVT or PE	Phase III/NI	Symptomatic or incidental VTE recurrence	Major bleeding	6

Abbreviations: CRNMB, clinically relevant nonmajor bleeding; DVT, deep-vein thrombosis; NI, noninferiority trial; PE, pulmonary embolism; S, superiority trial; VTE, venous thromboembolism.

In view of the available experience, several aspects should be considered to improve the homogeneity, relevance, and applicability of study results in the context of CT. These aspects include the type and the definition of active cancer, VTE qualification, and study methodology which are currently a source of heterogeneity.

Patients with cancer are highly diverse given the differences in age, comorbidities, and cancer sites and related antineoplastic treatments. The recruitment of patients with a homogeneous profile especially on the cancer type may be considered to improve the relevance and reproducibility of study results.

The two possible definitions of active cancer are summarized in Table 2 . The “broad definition” 36 is used in most CT trials but up to 25% of patients have their cancer “cured” at the time of the index VTE event. The “restrictive definition” 24 may reflect more accurately the condition at higher risk of VTE recurrence thus defining the cancer as “truly” active. From a feasibility viewpoint, the “broad definition” is likely to facilitate patient recruitment as opposed to “restrictive definition.”

**Table 2:** Definitions of active cancer in patients to be included in cancer-associated thrombosis trials

	Features defining active cancer
Broad definition 36	• Cancer diagnosed within the previous 6 months a or • Recurrent, regionally advanced, or metastatic cancer or • Cancer for which treatment had been administered within 6 months a or • Hematological cancer that is not in complete remission
Restrictive definition 24	• Cancer has not received potentially curative treatment or • There is evidence that treatment has not been curative (e.g., recurrent or progressive disease) or • Treatment is ongoing

a In some trials, including HOKUSAI-VTE Cancer, 21 this item becomes “cancer diagnosed within the previous 2 years.”

Main study features to be considered for trial design are summarized in Table 3 . Preferred recommended options for study relevance may include (1) the selection of symptomatic VTE events with stratification when both symptomatic and incidental events are considered as efficacy endpoints, (2) the choice of recurrent VTE as the primary efficacy endpoint, and (3) phase III trials design to ensure statistical power and conclusive results.

**Table 3:** Study features and questions relevant to the applicability of CT trial results

Questions	Suggested options
Active cancer definition: broad or restricted?	Restricted definition
VTE qualification: symptomatic, incidental, or both?	Symptomatic If both, stratification at randomization Investigate for symptoms in case of incidental PE
Detailed VTE qualification: Spontaneous or postoperative DVT? Incidental DVT or PE? DVT distal to mechanical obstruction (tumor)? Catheter-related thrombosis? Ongoing antineoplastic treatment at inclusion?	
All cancers or single type of cancer?	Only one type of cancer
Study methodology Trial type: phase III, phase II, pilot, or cohort? Primary endpoint: recurrent VTE, bleeding, or composite of both? Endpoint assessment after clearly defined treatment duration Outcomes: symptomatic or symptomatic + incidental events? Per protocol results available? Data of concomitant anticancer treatment available?	Phase III Recurrent VTE 6 mo Symptomatic events

Abbreviations: CT, cancer-associated thrombosis; DVT, deep-vein thrombosis; PE, pulmonary embolism; VTE, venous thromboembolism.

### Manuscript Limitations

Unlike usual reviews, our manuscript was limited to the review and discussion of one study. Only properly designed phase III trials are likely to generate relevant and conclusive data and that is the reason why we have concentrated our analysis on HOKUSAI-VTE Cancer. The meta-analysis by Li et al 33 on Select-D 23 and HOKUSAI-VTE Cancer 21 resulted in similar findings compared with HOKUSAI-VTE Cancer and no further randomized controlled studies are available to date to extend the field for another meta-analysis. Nevertheless, this review, even though limited to one trial, is consistent with our objectives to raise methodological issues to be addressed in view of improving the design of future studies.

## Conclusions

The HOKUSAI-VTE Cancer study is an important contribution to the evaluation of DOACs for the treatment of patients with CT. Even though edoxaban was noninferior to dalteparin on the composite endpoint of VTE recurrence and major bleeding, the usefulness of edoxaban in CT patients raised some concerns since it was associated with a significant increase in the risk of major bleeding compared with dalteparin especially in patients with GI cancer. In fact, the choice of a composite primary endpoint combining time to VTE recurrence or major bleeding and the study positivity for noninferiority suggested that the benefit–risk ratios of both agents was comparable at 12 months. The loss of power between 6 and 12 months of follow-up as well the inclusion of nearly 25% of patients who had cancer no longer makes it difficult to draw clear and definite conclusions for all patients with CT. The issue of the difference in anticoagulation regimens (LMWH or DOACs) for different types of cancer requiring an individualized approach of risks and benefits to tailor the management of CT remains unaddressed. The experience with the HOKUSAI-VTE Cancer study is likely to cause an important shift in the approach of the anticoagulant treatment of patients with CT. Future trials shall take in account relevant features such as the cancer type and activity, the underlying antineoplastic treatment, symptomatic events, age, and comorbidities, while efficacy and safety endpoints should be definitively separated. This would allow a more reliable assessment of the clinical benefit of the therapy in the target population in the context of future trials. Further research in this complex setting is warranted.
