# Erratum: Treatment of Cancer-Associated Thrombosis: Beyond HOKUSAI

**DOI:** 10.1055/s-0039-3400276

**Published:** 2019-11-05

**Authors:** Isabelle Mahé, Ismaïl Elalamy, Grigoris T. Gerotziafas, Philippe Girard

**Affiliations:** 1 Université de Paris, Innovations Thérapeutiques en Hémostase, INSERM, Paris, France; 2 Service de médecine Interne, AH-HP, Hôpital Louis Mourier, Colombes, Université de Paris, France; 3 F-CRIN INNOVTE, Saint Etienne, France; 4 Hematology and Thrombosis Center, Tenon University Hospital, Sorbonne University, INSERM U938, Paris, France; 5 Department of Obstetrics and Gynecology, The First I.M. Sechenov Moscow State Medical University, Moscow, Russia; 6 Research Group “Cancer, Haemostasis and Angiogenesis,” INSERM UMR_S 938, Centre de Recherche Saint-Antoine, Faculty of Medicine, Institut Universitaire de Cancérologie, Sorbonne Universities, Paris, France; 7 Service d'Hématologie Biologique Hôpital Tenon, Hôpitaux Universitaires de l'Est Parisien, APHP.6, Paris, France; 8 Institut du Thorax Curie-Montsouris, l'Institut Mutualiste Montsouris, Paris, France

It has been brought to the publisher's attention, that there was an error in Table 1 of the above article, published in *TH Open* , Volume 3, Number 3, 2019, pages e309–e315 (DOI: 10.1055/s-0039-1696659 ). In the SELECT-D study, it should state “ *N* = 406” instead of “ *N* = 203”.

The correct table appears below.

**Table 1:** Protocol design of randomized control trials on anticoagulants for the treatment of cancer-associated thrombosis

Study (year)	Study drug/comparator	Patients	Methods/statistics	Primary endpoint	Secondary endpoints	Duration (mo)
ONCENOX ^39^ *N* = 122 (2006)	Enoxaparin/warfarin	Active cancer, acute symptomatic VTE	Pilot feasibility	VTE recurrence	Major and minor bleeding	6
CANTHANOX ^25^ *N* = 146 (2002)	Enoxaparin/warfarin	Active or treated cancer, PE, and/or DVT	S	Composite of major bleeding or recurrent VTE	Recurrent VTE Major bleeding	3
CLOT ^26^ *N* = 672 (2003)	Dalteparin/warfarin	Active cancers, acute proximal symptomatic DVT or PE	Phase III/S	Symptomatic recurrence of DVT, PE, or both	Major bleeding Any bleeding	6
LITE CANCER ^27^ *N* = 200 (2006)	Tinzaparin/warfarin	Cancer, acute proximal DVT	Phase III/S	Recurrent VTE or death	Major and minor bleeding	3
CATCH ^22^ *N* = 900 (2015)	Tinzaparin/warfarin	Active cancers, acute proximal symptomatic DVT or PE	Phase III/S	VTE recurrence: proximal DVT, PE either symptomatic or incidental	Major bleeding CRNMB	6
SELECT-D ^23^ *N* = 406 (2018)	Rivaroxaban/dalteparin	All active cancers, proximal DVT, PE, incidental PE	Pilot	VTE recurrence: proximal DVT, PE either symptomatic or incidental, and other sites	Major bleeding CRNMB	6
HOKUSAI-VTE Cancer ^21^ *N* = 1,046 (2018)	Edoxaban/dalteparin	Active or history of cancer, acute symptomatic or incidental VTE	Phase III/NI	Composite of VTE recurrence (symptomatic or incidental) or major bleeding	VTE recurrence Major bleeding CRNMB	12
CARAVAGGIO ^35^ *N* = 1,168 (2019)	Apixaban/dalteparin	Active or history of cancer, symptomatic or incidental proximal DVT or PE	Phase III/NI	Symptomatic or incidental VTE recurrence	Major bleeding	6

Abbreviations: CRNMB, clinically relevant nonmajor bleeding; DVT, deep-vein thrombosis; NI, noninferiority trial; PE, pulmonary embolism; S, superiority trial; VTE, venous thromboembolism.

